# Efficacy and Safety of an Oxalic Acid and Glycerin Formulation for *Varroa destructor* Control in Honey Bee Colonies During Summer in a Northern Climate

**DOI:** 10.3390/pathogens14080724

**Published:** 2025-07-22

**Authors:** Daniel Thurston, Les Eccles, Melanie Kempers, Daniel Borges, Kelsey Ducsharm, Lynae Ovinge, Dave Stotesbury, Rod Scarlett, Paul Kozak, Tatiana Petukhova, Ernesto Guzman-Novoa, Nuria Morfin

**Affiliations:** 1 Ontario Technology Transfer Program, Ontario Beekeepers’ Association, Guelph, ON N1H 6J2, Canada; 2 Buzzworthy Solutions, Lethbridge, AB T0L 0V0, Canada; 3 Canadian Honey Council, Sherwood Park, AB T8E 1H1, Canada; 4 Ontario Ministry of Agriculture, Food, and Agribusiness, Guelph, ON N1G 4Y2, Canada; 5 Department of Population Medicine, University of Guelph, Guelph, ON N1G 2W1, Canada; 6 School of Environmental Sciences, University of Guelph, Guelph, ON N1G 2W1, Canada; 7 Department of Entomology, University of Manitoba, Winnipeg, MB R3T 2N2, Canada

**Keywords:** *Varroa destructor*, integrated pest management, acaricide, varroosis, *Apis mellifera*, honey bees, oxalic acid

## Abstract

Effective control of the parasitic mite *Varroa destructor* in honey bee (*Apis mellifera*) colonies relies on integrated pest management (IPM) strategies to prevent mite populations from reaching economic injury levels. Formulations of oxalic acid combined with glycerin may provide a viable summer treatment option in continental Northern climates. This study evaluated the efficacy of oxalic acid and glycerin strips compared to oxalic acid dribble and 65% formic acid when applied in mid-August. Mite levels and colony health parameters were assessed, and honey samples from oxalic acid-treated colonies were analyzed for residue levels. Results showed that the oxalic acid and glycerin strips had a moderate acaricidal efficacy (55.8 ± 3.2%), which was significantly higher than those of 65% formic acid (42.6 ± 3.2%) and oxalic acid dribble (39.5 ± 4.3%), which did not differ between them, suggesting potential for summer mite control. No significant adverse effects on cluster size, worker mortality, queen status, or colony survival were observed. Oxalic acid and glycerin increased the proportion of spotty brood patterns at early timepoints after treatment, but recovery was noted after 45 days of starting the treatment. Similar effects on brood were observed with 65% formic acid 14 days after starting the treatment, with recovery by 28 and 45 days after starting the treatment. No significant differences in oxalic acid residues in honey from the control and treatment colonies were found. Oxalic acid and glycerin strips might help control varroa mite populations, delaying their exponential growth and helping reduce economic losses for beekeepers, but this treatment should be considered as part of an IPM strategy and not a stand-alone method for *V. destructor* control.

## 1. Introduction

The ectoparasite *Varroa destructor* is the primary cause of honey bee (*Apis mellifera*) colony mortality in North America [1,2]. *Varroa destructor*, originally from Asia, has now spread globally, demonstrating an ability to adapt to different environments [3,4]. *Varroa destructor* is a highly prevalent mite that vectors virulent viruses, such as deformed wing virus variant B [5]. Varroa mites cause immunosuppression, shorten bee lifespan, and reduce colony productivity, and parasitized colonies can collapse rapidly if no treatment is applied [2,6,7]. Therefore, beekeepers should use integrated pest management (IPM) strategies to monitor varroa mite levels and intervene with cultural and mechanical methods to reduce mite populations and possibly apply acaricidal treatments to lower the risk of varroa mites reaching injury levels [8]. In many regions of Canada, such as the provinces of Ontario and British Columbia, the economic threshold for varroa mite infestation levels in honey bee colonies is 3% [9,10]. However, a recent revision of the economic injury levels of varroa mites indicated that colonies with a ≥1% mite infestation rate during late summer or early fall are more likely to die in the spring than colonies with lower rates of mite infestation [11]. However, it is challenging for beekeepers to maintain varroa mite levels below 1%, because the mite population grows as bee brood increases, given that its population depends on the honey bee colony’s life cycle [12]. The reproductive cycle of *V. destructor* occurs inside the capped cells of honey bee brood. The number of reproductive mites increases when bee brood is present in the colony (spring and summer), followed by a noticeable rise in adult mites during late summer and early fall in temperate climates [12]. Therefore, it is in beekeepers’ best interest to reduce mite levels during the summer to prevent mite populations from reaching injury levels in the fall. However, the number of acaricides registered for use during the summer is limited. For example, in Canada, formic acid and HopGuard 3^®^ are registered for use during the honey flow [13]. Complementary treatments, such as thymol (e.g., Thymovar^®^) and oxalic acid (dribbled or vaporized), can be applied in the spring or fall to help reduce varroa mite levels [13] but not when honey supers are present. Additionally, synthetic acaricides such as amitraz, tau-fluvalinate, and flumethrin have been used by beekeepers for decades, raising concerns about reduced efficacy and the development of resistance in mites [14,15]. These treatments also cannot be used when honey supers are present [13].

Oxalic acid is widely used for varroa mite control in North America, mostly for late fall treatment of colonies, as a 2–3% solution in sugar syrup (also known as the oxalic acid dribble, drizzle, or tickle method) or delivered with a vaporizer. The oxalic acid dribble method consists of applying the sucrose solution containing oxalic acid directly onto the bees in the hive’s brood chamber, whereas the vaporizing method consists of heating oxalic acid crystals with an electric device, which releases vapors from the chemical in the interior of the hive. In addition to this, an alternative method for varroa mite control that has been approved in some countries, like Argentina and Spain, consists of mixing oxalic acid and glycerin, and delivering the solution embedded on cellulose strips [16]. Commercial products based on this method are also available in some countries like Uruguay and the United Kingdom (Varroxan^®^, Vita Bee Health; Basingstoke, UK) with manufacturers claiming a miticidal efficacy of >90%.

The varroacidal activity of oxalic acid is related to its acidic nature, although the specific mode of action remains unknown [17]. This chemical can kill mites during their dispersal phase, but unlike formic acid it cannot penetrate wax cappings to kill mites inside cells [17]. To date, there are no records of varroa mites developing resistance to oxalic acid.

There is an increasing need for acaricidal treatments that can be applied during summer to help reduce mite population growth. Studies on oxalic acid and glycerin formulations suggest that they do not contaminate honey and have moderate to no effect on brood and queens. However, most of these studies have been conducted in tropical and subtropical environments [16,18,19]. Regional variation may influence the efficacy of organic treatments, which are known to be sensitive to seasonal factors such as temperature and humidity [20]. To date, only two peer-reviewed studies have evaluated the efficacy of an oxalic acid–glycerin formulation as a fall treatment in northern climates [21,22], and only one study has tested its application during the summer [23]. Thus, the objectives of this study were (a) to evaluate the efficacy of an oxalic acid–glycerin formulation for controlling varroa mite population growth in the summer in a continental climate characterized by cold winters and humid summers; (b) to compare the efficacy of the formulation with that of registered formulations, including oxalic acid dribble and 65% formic acid applied during the same summer period; (c) to assess the effects of the treatments on bee cluster size, worker mortality, brood pattern, queen status, and colony survival; and (d) to test for oxalic acid residues in honey from control colonies and colonies treated with the oxalic acid–glycerin formulation.

## 2. Materials and Methods

### 2.1. Experimental Setup and Acaricide Treatment

The experimental trials were conducted in three apiaries located in Guelph, Ontario, Canada (43°32′59.9964″ N and 80°15′0.0000″ W). One hundred and sixteen colonies were established and managed in single brood chamber Langstroth hives, using locally sourced queens. A compromised power analysis using a two-tailed test demonstrated that the total sample size of 116 colonies achieved a power of 0.997 (1-β) with an α value of 0.05 An ideal power is considered between 0.8 and 0.9 [24]; thus, the study had enough colonies to conduct the analyses. The experimental hives were fitted with screened bottom boards to facilitate the placement of a sticky paper on each of them to capture naturally fallen mites. Prior to the trials, the colonies were equalized in strength as per Guzman-Novoa et al. [25]. The 116 colonies were randomly assigned to each of three experimental apiaries and to each of the four treatments: oxalic acid and glycerin strips (*n* = 29), oxalic acid dribble (*n* = 29), 65% formic acid (NOD, Trenton, ON, Canada; *n* = 29), and control (no treatment; *n* = 29).

**Figure 1:**
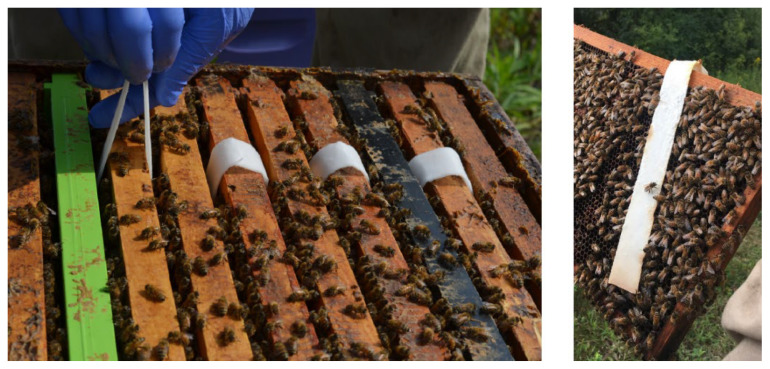
Four cellulose strips (24 cm × 4 cm) containing approximately 10 g of oxalic acid were hung over the top bars of four frames (one per strip) vertically of the brood chamber of each colony and left for 42 days.

The experimental trials began in mid-August 2018 (with daily maximum temperatures ranging from 20 to 34 °C during the treatment periods). Seasonal transitions and climatic variability in continental climates differ significantly from tropical or Mediterranean beekeeping regions, including floral ecosystems and humidity levels [26]; thus, it was considered crucial to test the product in northern conditions. Treatments were applied as follows: The oxalic acid and glycerin formulation was prepared by soaking four cellulose strips (24 cm × 4 cm) for 30 min in a solution of 40 g of oxalic acid dehydrate and 80 mL of glycerin (equivalent to 100.8 g of glycerin), as per Maggi et al. [16]. The mixture was gently heated in a water bath until a homogeneous solution was obtained before immersing the strips in it at 40 °C. Each strip absorbed approximately 10 g of oxalic acid, which was verified by observing that no solution was left in the container and by weighing the strips. The strips were kept in plastic containers until use; polyethylene is resistant to oxalic acid [27]. Four strips were hung over the top bars of four frames (one per strip) vertically between brood frames of the brood chamber of each colony as per Maggi et al. [16] (Figure 1) and remained inside the hives for 42 days. The aim of this study was to evaluate the effect of the treatment in single brood chambers in equalized colonies (same strength of adult bees and brood).

For the oxalic acid dribble method, a 3.5% oxalic acid solution was prepared by dissolving 35 g of oxalic acid crystals in 1 L of 50% sucrose syrup. A total of 50 mL of the solution was dribbled between the frames of each hive using a syringe. This treatment was applied six times at 4-day intervals, resulting in a total treatment of 10.5 g of oxalic acid per colony.

Pads with formic acid were prepared by pouring 20 mL of 65% formic acid onto each pad, as per the manufacturer’s instructions (NOD Apiary Products, Trenton, ON, Canada). Two pads were placed above the brood chamber frames of each colony every four days during six occasions, for a total treatment of 240 mL of formic acid per colony.

### 2.2. Colony Evaluations

To evaluate the efficacy of the acaricides, the daily mean of fallen varroa mites on sticky papers was determined before treatments (the week before applying treatments), during treatments, and after the final treatments. During the first seven days of treatments, all colonies were monitored daily by counting mites on sticky papers. After that period, sticky papers were placed and collected from the hives every three days to calculate the average number of mites that fell in 24 h. After 42 days of applying the first treatment, amitraz strips (Apivar^®^; Véto Pharma, Palaiseau, France) were placed in the hives as a finisher treatment for 42 days [21] according to the manufacturer’s recommendations. Prior to the trials, Pettis tests with modifications as per Eccles et al. [28] were conducted to confirm that varroa mites were not resistant to amitraz. The miticide efficacy of the amitraz strips coincided with the 90–97% efficacy reported by Morfin et al. [29] in apiaries from Ontario, Canada.

The miticide efficacy of each treatment was determined using the following equation, as per Sabahi et al. [21]:

Efficacy (%) = ∑[N_1_/(N_1_ + N_2_)] × 100

where N_1_ is the number of varroa mites that fell on the sticky papers during the 42 days of treatment and N_2_ is the number of mites that fell during the final treatment with amitraz.

The cluster size of the colonies was evaluated as per Nasr et al. [30] after the 42-day treatment with oxalic acid and glycerin strips. Briefly, the lid of each hive was opened to inspect the frames in the brood chamber and locate the bee cluster from above. The number of frames fully or partially covered with bees was recorded. Then, the box was tilted backwards to observe and record the number of frames covered with bees from below. The top and bottom cluster size of each colony was averaged and calculated as the number of frames covered by bees, rounded to the nearest half frame. This procedure was performed early in the morning, before bee foraging started.

To assess the effect of the acaricide treatments on worker bee mortality, Todd’s dead bee traps [31] were installed at the entrance of hives for the duration of the treatments, and dead brood (larvae and pupae) and adult bees in the interior of the traps were counted weekly.

The queen status and brood pattern of each colony were assessed at four time points (22 and 29 August and 12 and 27 September 2018). Brood patterns were arbitrarily classified as spotty or uniform. The brood pattern of a colony was classified as spotty if at a visual inspection performed by two observers >20% of the brood area of the combs evaluated had empty cells scattered and interspersed. Alternatively, a colony’s brood pattern was classified as uniform if at a visual inspection <20% of the combs’ brood area had empty cells [32]. Queen status was classified as satisfactory or unsatisfactory: unsatisfactory if supersedure or emerging cells were observed or if no eggs or multiple eggs inside comb cells were noted based on visual inspections. The queen status was classified as satisfactory if no supersedure or emerging cells were observed and if one egg per cell was noted during visual inspections. Additionally, the number of dead, alive, and weak colonies was recorded in the spring of 2019.

Lastly, honey samples were collected from the supers of colonies treated with oxalic acid and glycerin strips, as well as from the control group at three time points: pre-treatment (31 July), mid-treatment (5 September), and post-treatment (27 September). Honey samples were stored at 4 °C until analysis. Oxalic acid residues were measured with an Oxalic Acid Colorimetric Kit (Sigma-Aldrich, St. Louis, MO, USA) according to the manufacturer’s instructions, using 10 µL of each honey sample within seven days of collection.

### 2.3. Statistical Analyses

The data were subjected to Shapiro–Wilk and Levene tests to assess for normality and homogeneity of variance. The data that did not comply with the two assumptions were rank transformed before being subjected to non-parametric tests. To justify the sample size used in the analyses, a compromise or post hoc power analysis was done with the number of colonies in each treatment as per Erdfelder et al. [33]. The data on efficacy, cluster size, and bee mortality were analyzed with Kruskal–Wallis tests and Conover–Iman procedures to determine if there were significant differences between treatments, and were subjected to aligned rank-transformed ANOVAs to determine the effects of the apiary and the treatment on each response variable Additionally, contingency tables were used to analyze the effect of oxalic acid and glycerin on brood pattern. A Fisher’s exact test was used to assess the effect of the treatments on the proportion of colonies showing queen unsatisfactory status, and the proportion of dead, alive, and weak colonies in the spring following the trials. A Friedman test was used to analyze the effect of the treatments on the number of dead worker bees (adults and brood) found in the traps. Lastly, the Kruskal–Wallis test and Dunn’s procedure with Bonferroni adjustment after rank transformation were used to determine differences for the concentration of oxalic acid residues between honey samples from colonies treated with oxalic acid and glycerin strips and the control, and between collection timepoints. The statistical analyses were conducted using XLSTAT Lumivero (2024), R version 2023.12.1.102, G*Power 3.1.9.7 [34], and SAS^®^ Institute Inc. (Cary, NC, USA), with the significance level set at *p* < 0.05 (α of 0.05).

## 3. Results

### 3.1. Acaricide Treatment Efficacy

**Figure 2:**
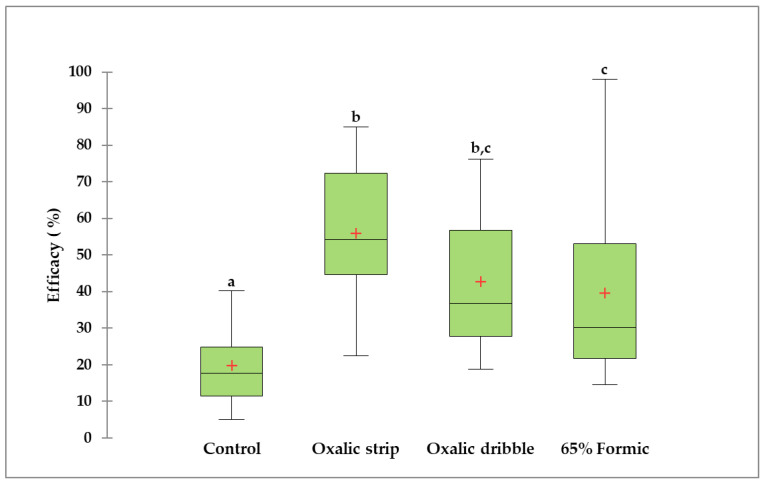
Percent varroacide efficacy of oxalic acid and glycerin strips, oxalic acid dribble, and 65% formic acid, plus control treatments. Medians are depicted with horizontal lines inside the boxes, and the 25th and 75th percentiles are shown as the bottom and upper lines of the boxes. The minimum and maximum values are represented by the vertical bars below and above the boxes. Means are shown as a red cross (+) inside the boxes. Non-transformed values are presented. Different letters above the boxplots indicate significant differences between treatments based on Kruskal–Wallis tests and Conover–Iman procedures (*p* < 0.0001; α of 0.05).

The efficacy scores of the three acaricide treatments were significantly higher than that of the control group (F = 47.49, *p* < 0.0001, df = 3; Figure 2 and Table S1). The treatment of oxalic acid and glycerin strips (55.8 ± 3.2%) was significantly more efficacious than the 65% formic acid (39.5 ± 4.3%) and the control treatments but did not differ from the oxalic acid dribble treatment (42.6 ± 3.2%; *p* < 0.01). The maximum efficacy found in oxalic acid plus glycerin treated colonies was 85.07%. Additionally, the variable apiary had no significant effect on treatment efficacy (F = 0.21, *p* = 0.81. df = 2).

### 3.2. Effects of Acaricides on Cluster Size

**Figure 3:**
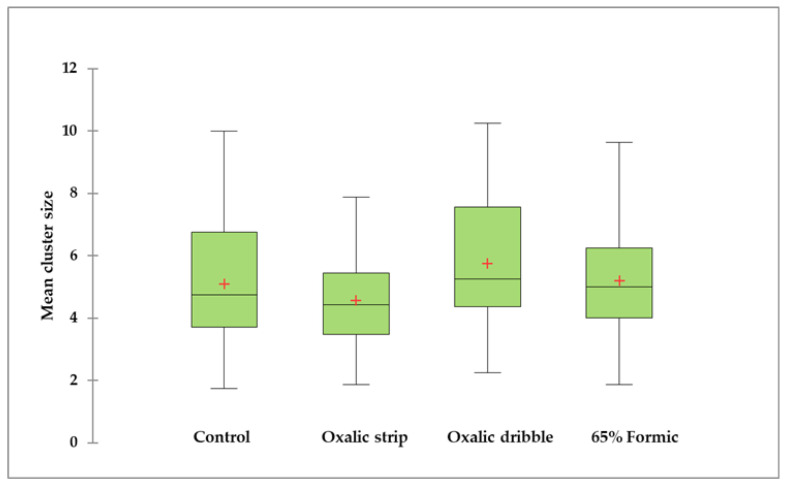
Mean cluster size of control colonies, and oxalic acid and glycerin strips, oxalic acid dribble, and 65% formic acid treated colonies. Medians are depicted with horizontal lines inside the boxes, and the 25th and 75th percentiles are shown as the bottom and top of the boxes. The minimum and maximum values are represented by the vertical bars below and above the boxes. Means are shown as a red cross (+) inside the boxes. Non-transformed values are presented. No significant differences between treatments were observed based on Kruskal–Wallis tests and Conover–Iman procedures (*p* > 0.05; α of 0.05).

The mean cluster size of each colony was calculated by averaging the top and bottom cluster sizes at the end of the trials. The worker cluster sizes were not significantly different between treatments (K = 4.44, df = 3, *p* = 0.218; Figure 3 and Table S2). Additionally, the apiary variable had no significant effects on cluster size (F = 0.91, *p* = 0.40, df = 2).

### 3.3. Effects of Acaricides on Worker Bee Mortality

No significant differences were found for the number of dead worker bees between treatments (K = 2.12, df = 3, *p* = 0.54; Supplementary Table S3). Similarly, no differences in the number of dead larvae and pupae in traps of the different treatments were found during six weeks of observations (K = 1.18, df = 3, *p* = 0.75; Table S3). Additionally, the apiary variable had no significant effect on the number of dead adult bees or brood (F = 0.47, *p* = 0.62, df = 2; F = 1.73, *p* = 0.18, df = 2, respectively).

### 3.4. Effect of Acaricides on Brood Patterns

The oxalic acid with glycerin treatment had a significant effect on the proportion of colonies with spotty brood pattern at 7, 14, and 28 days after starting the treatment (*p* < 0.05; Table 1). However, the brood pattern appeared to recover based on observations 45 days after starting the treatment (*p* > 0.5). Effects on brood pattern were also observed in colonies treated with 65% formic acid 14 days after starting the treatment (*p* < 0.05), but a brood pattern recovery was also observed two weeks later (28 and 45 days after starting the treatment) (*p* > 0.05). The control and oxalic acid dribble treatments did not show significant effects on the proportion of colonies with spotty brood patterns at any time point (*p* > 0.05).

### 3.5. Effects of Acaricide Treatments on Queen Status

No significant differences for the proportion of colonies with unsatisfactory queen status were found between treatments 7, 14, and 28 days after treatment (*p* > 0.05; Table 2).

### 3.6. Effects of Acaricide Treatments on Colony Mortality

No significant differences between treatments were found for colony mortality rate (0.38, 0.21, 0.26, and 0.24, for the control, oxalic acid with glycerin, oxalic acid dribble, and 65% formic acid treatments, respectively; *p* = 0.51). Similarly, no differences were observed between treatments in the spring of 2019 for the proportions of colonies classified as dead, alive, or weak in the spring of 2019 (*p* = 0.18; Table 3).

### 3.7. Oxalic Acid Residues in Honey

The mean concentration of oxalic acid in the control colonies was 36.85 µg/g (±6.68) and 39.36 µg/g (±6.74) in the oxalic acid- and glycerin-treated colonies. No significant differences in concentration of oxalic acid residues in the honey from colonies treated with oxalic acid and glycerin strips and in the honey from the control colonies were found in the samples taken before the treatment (K = 3.84, df = 1, *p* = 0.47; Table S4) and during the treatment (K = 3.84, df = 1, *p* = 0.87; Table S4), but a significantly lower content of oxalic acid was noted in honey collected from colonies treated with oxalic acid compared to the control after the treatment (9.87 ± 0.87 and 16.66 ± 4.18, respectively; K = 3.84, df = 1, *p* = 0.044; Table S4).

## 4. Discussion

This study showed that the formulation of oxalic acid and glycerin in strips applied during summer achieves a moderate level of varroa mite control in honey bee colonies (55.8 ± 3.2% efficacy). This efficacy level may not be optimal, but reducing mite levels in the summer can significantly slow down the exponential growth of varroa mite populations, and when used as part of an IPM strategy it may prevent varroa mite levels from reaching economic injury levels early in the fall [35]. In continental northern climates, including Canadian provinces, beekeepers must apply acaricidal treatments immediately after honey flow ends, which is a short time window for overwintering colonies. Considering that colonies with ≥1% varroa infestation are more likely to die during winter compared to colonies with <1% mite infestation [11], achieving even a 50% reduction in mite populations should be regarded as valuable control of an IPM strategy. Rather than aiming for complete mite eradication, IPM strategies focus on regulating mite populations to maintain them below economic injury levels. Thus, an IPM strategy for varroa mite control incorporates multiple tools, including honey bee stock selected for *V. destructor* resistance, cultural and mechanical methods of mite removal, and the use of organic and synthetic acaricides [8]. Acaricide formulations with moderate efficacy, such as the one reported in this study, should not be used as a stand-alone treatment for *V. destructor* control, but rather as part of an integrated pest management (IPM) strategy. Ideally, an IPM strategy should aim at restraining varroa mite population growth during the summer through the application of organic acaricides. The effectiveness of organic acaricides depends on factors such as nectar flow and the colony’s life cycle stage (e.g., broodless periods), as well as environmental conditions [20]. Therefore, finding formulations that can help deliver organic acaricides is imperative for effectively reducing mite populations, particularly when ambient temperatures and humidity are high, like in the summer months.

Formulations of oxalic acid and glycerin have shown promising results for fall treatments. For example, a previous study conducted in Ontario Canada reported varroacidal efficacy of 78.7 ± 3.9% for a formulation containing 6 g of oxalic acid and 6.5 mL of glycerin per strip [21]. This efficacy rate is higher than the one reported in this study, which indicates that higher varroacidal efficacy could be achieved in the fall with less active ingredient per strip. These differences in efficacy could be related to differences in climatic conditions and amounts of brood in the colonies between the seasons. During the fall, there is less brood in the colonies than in the summer, and most varroa mites are in their dispersal phase, making them more vulnerable to oxalic acid exposure, as oxalic acid cannot penetrate capped cells [17]. This could explain the higher efficacy of oxalic acid treatments (i.e., dribbled or vaporized) observed in the fall, which ranged from 65.3% to 97%, compared to summer applications [36,37,38].

The fall season in Ontario is characterized by lower ambient temperatures and humidity compared to the summer, and honey bee colonies have less capped brood in the fall than in the summer. Like other organic treatments, climatic conditions could play a role in the efficacy of oxalic acid with glycerin within the hive [38]. Pamondon et al. [22] reported an efficacy of 20.7% in colonies treated during the summer with 27 g of oxalic acid and glycerin in a continental climate. Similarly, Hristov et al. [23] reported an efficacy of 17.05% under comparable climatic conditions using strips with a 33% concentration of the active ingredient. However, Maggi et al. [16] reported an average acaricidal efficacy of 93.1% for a formulation of 10 g of oxalic acid and glycerin in cellulose strips (Aluen CAP; Cooperativa de Trabajo Apícola Pampero, Buenos Aires, Argentina) during the summer in a subtropical environment in Argentina. A study conducted in the Gulf of Mexico tested the same formulation, finding a similar efficacy rate (87.8%) for colonies treated in the summer [18]. Similarly, Varroxsan^®^ (Apícola Integral, Progreso, Urugay), a registered product with comparable formulation (7 g of oxalic acid per cellulose strip), showed acaricidal efficacies against varroa mites of 96.5% and 83.9% when applied in the winter and spring, respectively. These trials were also conducted in a humid subtropical climate [19]. Additionally, the combination of 6 g of oxalic acid and glycerin in cellulose strips was recently tested under fall Mediterranean conditions. This 4-year study reported optimal acaricidal efficacies between 90.4 and 94.5% [39]. Conversely, a study conducted in southern Georgia, with a humid subtropical climate, found no significant reduction in mite levels between the control colonies and colonies treated with 12 and 18 g of oxalic acid and glycerin in cellulose strips in the summer [40]. Therefore, environmental conditions, including seasonal timing and regional climate, may influence the acaricidal efficacy of oxalic acid and glycerin delivered in cellulose strips. Our study found that the summer acaricidal efficacy of cellulose strips containing oxalic acid and glycerin (55.8 ± 3.2%) was significantly higher than that of a registered product containing 65% formic acid (NOD Apiary Products, Trenton, ON, Canada), which only achieved 39.5 ± 4.3% efficacy under the same conditions. Similarly, Plamondon et al. [22] found that 27 g of oxalic acid and glycerin strips had significantly higher efficacy compared to formic acid (Formic Pro Formic; NOD, NOD, Trenton, ON, Canada) (27% and 11%, respectively) applied in the summer. Formic acid is used by many beekeepers to reduce mite levels during the summer and is commonly included in IPM strategies. Formic acid and hop beta acids are the only two active ingredients allowed in formulations for varroa mite control that can be applied during honey flows in Canada (Formic Pro^®^ and Mite Away Quick Strips; NOD, NOD, Trenton, Ontario and HopGuard 3^®^; BetaTec, Washington, DC, USA). Oxalic acid dribble also showed moderate acaricidal efficacy in summer applications (42.6 ± 3.2%), indicating that both formulations with oxalic acid had similar results. Oxalic acid dribble is commonly applied in the fall as a finisher, complementary treatment (for example, used after the application of a synthetic acaricide).

Overall, the tested treatments of this study had minimal to no effects on the honey bee health parameters that were assessed, including colony strength, worker bee mortality (adults and brood), queen status, and colony mortality. Other studies have found no effects of oxalic acid and glycerin formulations on colony strength [16], number of dead worker bees in traps, and queen mortality [18,39]. However, the effects of oxalic acid have been found to significantly increase colony mortality [21] and our study also showed effects of oxalic acid and glycerin on brood patterns. The formulation significantly increased the proportion of colonies with spotty brood patterns 7 days after placing the oxalic acid strips, 14 days after placing the oxalic acid strips, and 28 days after placing the oxalic acid strips. Similarly, formic acid seemed to affect brood patterns 14 days after starting the treatment. But recovery, observed as a uniform brood pattern, was noted five weeks after treatment for both oxalic acid and glycerin strips and 65% formic acid. These observations are in line with anecdotal accounts of brood damage and queen failure after formic acid treatments. Thus, the effect of formic acid on brood patterns was not surprising and a relatively rapid recovery was noted. Repeated applications of oxalic acid vaporizations (20 g/colony) have not caused detrimental effects on queen acceptance and sperm quality [41], or on the number of dead brood [16,39,42]. However, a significant negative effect on larval removal after the application of oxalic acid dribble (3% *w*/*v* oxalic acid) has been reported [43].

Lastly, oxalic acid residues in honey were not significantly higher in colonies treated with the chemical relative to the control in samples collected before and during treatment which aligns with the findings of Maggi et al. [16], who also found no significant increase in oxalic acid residues in honey, bee samples, or beeswax of treated colonies compared with non-treated colonies. Also, Plamondon et al. [20] found no significantly higher residues of oxalic acid and of glycerin in honey from treated colonies compared to the control. A lower concentration of oxalic acid was found in honey samples from colonies treated with oxalic acid and glycerine relative to the control. Furthermore, none of the recorded concentrations of samples were above the normal content of oxalic acid naturally occurring in honey (11.3 to 160 µg/g) [44,45]. Oxalic acid is a naturally occurring organic acid in honey, and its levels may vary depending on the honey’s origin [44]. However, treatment with oxalic acid and glycerin at the doses assayed in this study does not appear to alter normal levels. Testing for acaricide residues is crucial to ensure that honey from treated colonies is safe for consumers. Although food grade glycerine was used in this study, we believe that further studies should test for glycerine residues in honey to ensure that hive products are safe for consumers. Nevertheless, Plamondon et al. [22] found no significant residues of glycerine in honey from colonies treated with oxalic acid and glycerine pads using a similar formulation containing one third oxalic acid and one third food grade glycerin.

In conclusion, this study demonstrates that oxalic acid and glycerin strips are relatively safe for honey bees, as no detrimental effects were observed on most health parameters evaluated. Additionally, the application method during the nectar flow season (summer) does not appear to compromise the quality of honey in treated colonies. Furthermore, oxalic acid and glycerin in cellulose strips applied to honey bee colonies in the summer exhibited moderate efficacy in controlling *V. destructor* infestations in continental Northern climates. Hence, when incorporated into an IPM strategy, this formulation could help reduce mite population growth and delay their exponential increase, preventing them from reaching economic injury levels.

## Figures and Tables

**Table 1:** Proportion of colonies showing a spotty brood pattern 7, 14, 28, and 45 days after starting the treatments with oxalic acid and glycerin strips, oxalic acid dribble, 65% formic acid, and the control. The asterisk indicates a significant increase in the proportion of spotty brood patterns based on Chi^2^ tests of independence and adjusted residuals (α of 0.05).

Treatment	Days After Treatment	Proportion of Colonies with Spotty Brood Patterns
Control	7	0.029
Oxalic acid strips	7	0.54 *
Oxalic acid dribble	7	0.029
65% formic acid	7	0.17
Control	14	0.097
Oxalic acid strips	14	0.69 *
Oxalic acid dribble	14	0.057
65% formic acid	14	0.69 *
Control	28	0.18
Oxalic acid strips	28	0.51 *
Oxalic acid dribble	28	0.23
65% formic acid	28	0.48
Control	45	0.50
Oxalic acid strips	45	0.12
Oxalic acid dribble	45	0.00
65% formic acid	45	0.37

**Table 2:** Proportion of colonies with an unsatisfactory queen status 7, 14, 28, and 45 days after starting the treatment with oxalic acid and glycerin strips, oxalic acid dribble, 65% formic acid, and the control. No significant differences in the proportions of colonies with unsatisfactory queen status were found between treatments based on Fisher’s exact tests (α of 0.05).

Treatment	Days of After Treatment	Proportion of Colonies with an Unsatisfactory Queen Status
Control	7	0.17
Oxalic acid strips	7	0.08
Oxalic acid dribble	7	0.00
65% Formic acid	7	0.20
Control	14	0.10
Oxalic acid strips	14	0.03
Oxalic acid dribble	14	0.03
65% Formic acid	14	0.10
Control	28	0.09
Oxalic acid strips	28	0.06
Oxalic acid dribble	28	0.09
Oxalic acid with glycerin	28	0.09
Control	45	0.10
Oxalic acid strips	45	0.06
Oxalic acid dribble	45	0.00
65% Formic acid	45	0.10

**Table 3:** Proportion of dead, weak, and live colonies recorded in the spring of 2019. No significant differences in the proportions of dead, weak, and live colonies treated with oxalic acid with glycerin, oxalic acid dribble, 65% formic acid, and the control were noted based on Fisher’s exact tests (α of 0.05).

Treatment	Proportion of Dead Colonies	Proportion of Live Colonies	Proportion of Weak Colonies
Control	0.38	0.38	0.24
Oxalic acid with glycerin	0.21	0.64	0.14
Oxalic acid dribble	0.26	0.70	0.04
65% Formic acid	0.24	0.59	0.17

## Data Availability

The raw data supporting the conclusions of this article is included in the Supplementary Files.

## Supplementary Materials

The following supporting information can be downloaded at https://www.mdpi.com/article/10.3390/pathogens14080724/s1, Table S1: Acaricide efficacy; Table S2: Cluster size; Table S3: Worker bees and larvae in dead traps; Table S4: Oxalic acid residues in honey.
